# Identification of putative causal associations between MicroRNAs and breast cancer via Mendelian randomization and bioinformatic analysis

**DOI:** 10.1097/MD.0000000000047494

**Published:** 2026-02-06

**Authors:** Jianlin Ren, Lifeng Zhu

**Affiliations:** a Department of Thyroid and Breast Surgery, Shaoxing Shangyu District Hospital of Traditional Chinese Medicine, Shaoxing, Zhejiang, China.

**Keywords:** breast cancer, cell cycle, Mendelian randomization, miRNA, quantitative trait loci

## Abstract

MicroRNAs (miRNAs) are implicated in breast cancer progression and prognosis. This study employed a Mendelian randomization (MR) framework to investigate causal relationships between plasma circulating miRNAs and breast cancer. miRNA expression quantitative trait loci were extracted from 2 independent cohorts. High-confidence miRNAs and their associated single-nucleotide polymorphisms were selected for 2-sample MR analyses using inverse-variance weighted and MR-Egger methods. Differential expression analysis and univariate Cox regression identified survival-associated genes in breast cancer, while enrichment analyses revealed pathways and biological processes linked to candidate targets. Pan-cancer analyses of miRNAs and targets were conducted via the ENCORI platform. Initial MR analyses in the discovery phase identified hsa-miR-100-5p, hsa-miR-125b-5p, and hsa-miR-339-5p as significantly associated with reduced breast cancer risk (*P* < .05), suggesting potential protective roles. A total of 1291 survival-associated differentially expressed genes were identified, with 39 overlapping targets implicated in miRNA-mediated breast cancer intervention. Enrichment analyses highlighted their involvement in cell cycle regulation and p53 signaling pathway. In the validation cohort, only hsa-miR-339-5p confirmed a protective effect on breast cancer risk, while hsa-miR-100-5p and hsa-miR-125b-5p did not reach significance. Pan-cancer profiling demonstrated aberrant miRNA expression across malignancies, prognostic relevance in multiple cancers, and significant negative correlations between miRNAs and target genes in breast tumors. Our findings provide novel insights into the causal roles of miRNAs in breast cancer pathogenesis and underscore their potential as noninvasive biomarkers and therapeutic targets. Future studies should prioritize functional validation and clinical translation of these miRNAs.

## 1. Introduction

Breast cancer stands as one of the most common malignancies among women globally, with its incidence and mortality rates continuing to rise in many countries.^[1]^ According to statistics from the World Health Organization, breast cancer has become a leading cause of cancer-related deaths among women.^[1]^ Despite significant advances in early screening and treatment methods that have notably improved survival rates for patients with breast cancer in recent years, numerous challenges remain, including disease heterogeneity,^[2]^ risk of recurrence,^[3]^ and resistance to existing therapies.^[4]^ Therefore, a deeper understanding of the mechanisms underlying breast cancer development and associated risk factors is crucial for devising effective prevention and treatment strategies.

MicroRNAs (miRNAs), a class of ~22-nucleotide noncoding RNA molecules, are widely involved in the regulation of gene expression. Research has demonstrated that miRNAs impact biological processes such as cell proliferation, differentiation, and apoptosis by targeting specific gene expressions.^[5]^ Accumulating evidence indicates that miRNAs play a pivotal role in the onset and progression of various cancers, including breast cancer.^[6]^ Specific miRNA expression profiles are closely associated with the occurrence, development, and prognosis of breast cancer, suggesting that miRNAs could serve as potential biomarkers and therapeutic targets.^[7,8]^ Recent studies have shown causal relationships between plasma circulating miRNAs and various diseases, including COVID-19,^[9]^ amyotrophic lateral sclerosis,^[10]^ and Parkinson disease,^[11]^ Additionally, several studies have explored associations between plasma circulating miRNAs and breast cancer, identifying potential diagnostic biomarkers for early detection through miRNA profiling and ratio analyses in screening cohorts.^[12–14]^ However, no prior research has applied a Mendelian randomization (MR) framework to investigate the causal links between plasma circulating miRNAs and breast cancer risk.

This study aims to investigate the causal relationship between miRNAs and breast cancer through the application of MR. Leveraging existing data on miRNA expression quantitative trait loci (eQTL) and genome-wide association studies (GWAS) of breast cancer, we will identify miRNAs significantly associated with breast cancer risk and analyze their potential targets and related pathways. Through this research, we hope to provide new insights and evidence for the early prevention and treatment of breast cancer.

## 2. Materials and methods

### 2.1. MiRNA eQTL data retrieval

Data on miRNA-eQTLs were obtained from 2 previous study cohorts. First, we utilized the cohort by Huan, T et al^[15]^ as the discovery cohort, which analyzed miRNA expression in whole blood samples of participants using quantitative reverse transcription PCR. This dataset included 280 high-quality miRNAs and ~10 million associated single-nucleotide polymorphisms (SNPs). In this study, we focused solely on cis-acting miRNA-eQTLs and further excluded SNPs located in coding regions with synonymous or missense consequences to mitigate potential pleiotropy effects. Ultimately, SNP instruments with a Benjamini–Hochberg corrected false discovery rate (FDR) < 0.1 were selected. Additionally, we acquired data from an additional miRNA eQTL validation cohort from Nikpay, M et al,^[16]^ encompassing miRNA expression levels in blood samples from 710 unrelated individuals of European ancestry. The miRNA IDs between the 2 datasets were harmonized using miRCarta v1.1.

### 2.2. Breast cancer GWAS data retrieval

GWAS data for breast cancer cases were sourced from the IEU open gwas project (https://gwas.mrcieu.ac.uk/, accessed on October 18, 2021), including data from 3 Consortia: MRC-IEU (GWAS ID: ukb-b-13584), Neale Lab (GWAS ID: ukb-a-213), and Breast Cancer Association Consortium (WAS ID: ieu-a-1126), comprising 35,102 cases and 388,356 controls, 25,865 cases and 283,784 controls, and 122,977 cases and case controls, respectively. All subjects were of European descent.

### 2.3. MR analysis

Two-sample MR analysis was performed using the R package TwoSampleMR v0.6.8,^[17]^ employing MR-Egger and inverse-variance weighted (IVW) methods to elucidate the genetic association between miRNAs and breast cancer. Linkage Disequilibrium (LD) clumping was conducted on IVs within the training set using *r*^2^ < 0.5 and a 10-kb window to identify independent IVs. Instrument strength was evaluated using F-statistics, with values >10 considered indicative of strong instruments to minimize weak instrument bias. SNPs not significantly associated with breast cancer (*P* > 5e−08) were retrieved from the breast cancer GWAS data. Exposure and outcome data were standardized to ensure common effect alleles, and palindromic SNPs were removed. An miRNA was considered causally related to breast cancer if it met the following criteria: *P*-value < .05 and FDR < 0.1 for the IVW test; consistent direction of estimated effect sizes across both models (IVW and MR-Egger); at least 3 SNPs present in the MR test; and high confidence in relationships claimed only when significant results are shown by IVW in the validation cohort. Heterogeneity was assessed using Cochran *Q* statistic derived from the IVW method, where a *P*-value > .05 indicates no significant heterogeneity. Directional horizontal pleiotropy was evaluated using the MR-Egger intercept test, with intercept values close to zero and *P* > .05 suggesting no evidence of bias. Leave-one-out analysis was employed to evaluate the stability of the causal estimates.

### 2.4. Identification of breast cancer-associated genes

Transcriptomic profiles, clinical data, and mutation signatures of the The Cancer Genomic Atlas (TCGA)-BRCA cohort were obtained from TCGA (https://portal.gdc.cancer.gov/). This cohort comprised 1113 breast cancer cases and 113 healthy controls. Differential expression analysis was performed using the limma package, with genes meeting the criteria of adjusted *P*-value < .05 and log2(fold change) > 1 considered statistically significant. Univariate Cox regression analysis was subsequently applied to screen differentially expressed genes (DEGs) significantly associated with overall survival (*P* < .05). Somatic mutation data were analyzed and visualized using the maftools package, and oncoplots were generated to display mutation landscapes of potential therapeutic targets in breast cancer.

### 2.5. miRNA target analysis

Experimentally validated miRNA–target interactions were identified using miRNet 2.0 (https://www.mirnet.ca/miRNet/home.xhtml; accessed on January 12, 2025),^[18]^ with supporting evidence sourced from the miRTarBase v9.0 database.^[19]^ A miRNA-gene regulatory network was constructed using Cytoscape v3.10.2. Venn analysis was performed to identify overlapping genes between miRNA targets and breast cancer-associated DEGs, which were defined as causal targets for miRNA-mediated intervention in breast cancer. Functional annotation of these overlapping genes was conducted via Gene Ontology and Kyoto Encyclopedia of Genes and Genomes pathway enrichment analyses using the ClusterProfiler v4.12.6 package.^[20]^ Significantly enriched terms were selected based on an FDR < 0.05, and results were visualized using dot plots.

### 2.6. Pan-cancer analysis

The ENCORI Pan-Cancer Analysis Platform (https://rnasysu.com/encori/) was employed for pan-cancer investigations. This platform enabled: expression comparisons between tumor and adjacent normal tissues across 17 cancer types; prognostic analysis across 32 cancer types; and co-expression analysis of miRNAs and their targets in 17 cancer types.

## 3. Results

### 3.1. Characteristics of miRNA eQTL data

**Figure 1.**
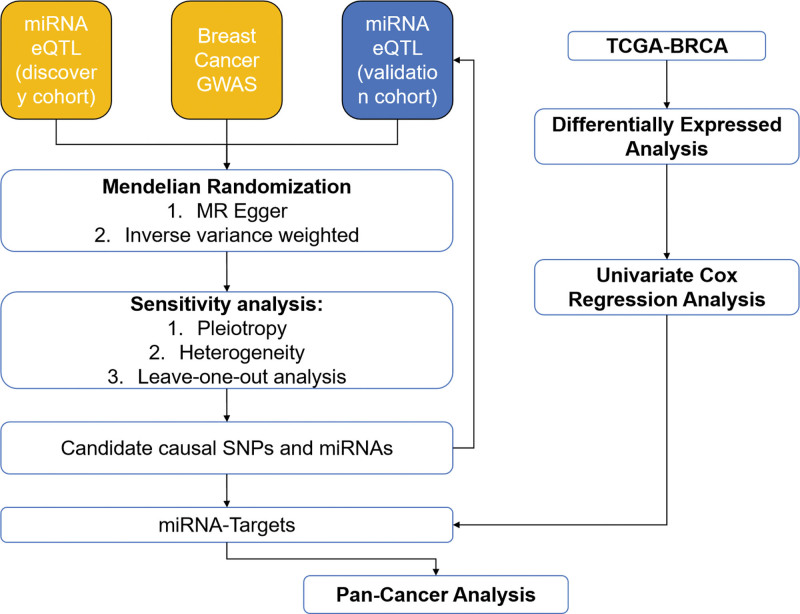
Flowchart of the study design. This flowchart illustrates the overall workflow, including data retrieval from miRNA eQTL cohorts (Huan et al^[15]^ as discovery cohort, n = 280 high-quality miRNAs and ~10 million SNPs; Nikpay et al^[16]^ as validation cohort, n = 2083 miRNAs and 164,336 SNPs at *P* < 1e−5), GWAS data from 3 consortia (MRC-IEU: GWAS ID ukb-b-13584, 35,102 cases/388,356 controls; Neale Lab: GWAS ID ukb-a-213, 25,865 cases/283,784 controls; and BCAC: GWAS ID ieu-a-1126, 122,977 cases/controls), MR analyses using IVW and MR-Egger methods, target identification using TCGA-BRCA cohort (1113 breast cancer cases/113 healthy controls), and pan-cancer analyses via the ENCORI platform. BCAC = Breast Cancer Association Consortium, eQTL = expression quantitative trait loci, GWAS = genome-wide association studies, IVW = inverse-variance weighted, MR = Mendelian randomization, SNP = single nucleotide polymorphism.

Initially, we preprocessed the eQTL data from Huan, T et al’s cohort, selecting 9500 SNPs out of 9612 through a process that excluded those directly associated with coding regions (to mitigate potential pleiotropy) and removed 17 SNPs related to mir-213 due to failure in mapping to valid miRNA IDs. This resulted in 9500 SNPs serving as instrumental variables for 75 miRNAs for MR analysis, and the selected SNPs demonstrated strong instrument strength, as evidenced by F-statistics > 10 for all key miRNAs (Table S1, Supplemental digital content, https://links.lww.com/MD/R299). Additionally, we compiled 164,336 SNPs (*P* < 1e−5) associated with 2083 miRNAs from Nikpay, M et al’s study for validation purposes. A flowchart illustrating the study design is provided in Figure 1.

### 3.2. Protective effects of plasma circulating miRNAs on breast cancer

**Figure 2.**
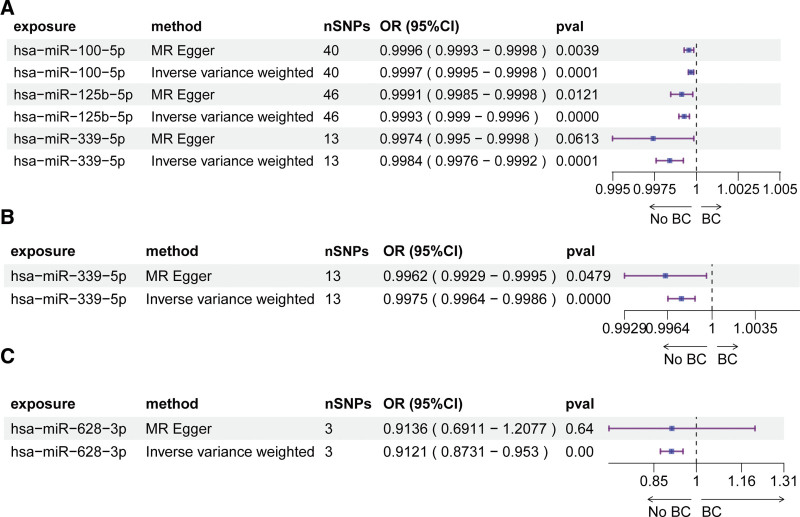
MR estimates for the causal relationship between plasma circulating miRNAs and breast cancer. Significant causal relationships identified in the (A) MRC-IEU consortium (GWAS ID ukb-b-13584, 35,102 cases/388,356 controls), (B) Neale Lab consortium (GWAS ID ukb-a-213, 25,865 cases/283,784 controls), and (C) BCAC consortium (GWAS ID ieu-a-1126, 122,977 cases/controls). Estimates derived from inverse-variance weighted (IVW) method; *P*-values from IVW test (*P* < .05) with FDR < 0.1; consistent effect directions across IVW and MR-Egger models; at least 3 SNPs per miRNA. BC = breast cancer, BCAC = Breast Cancer Association Consortium, CI = confidence interval, FDR = false discovery rate, MR = Mendelian randomization, OR = odds ratio, SNP = single nucleotide polymorphism.

**Figure 3.**
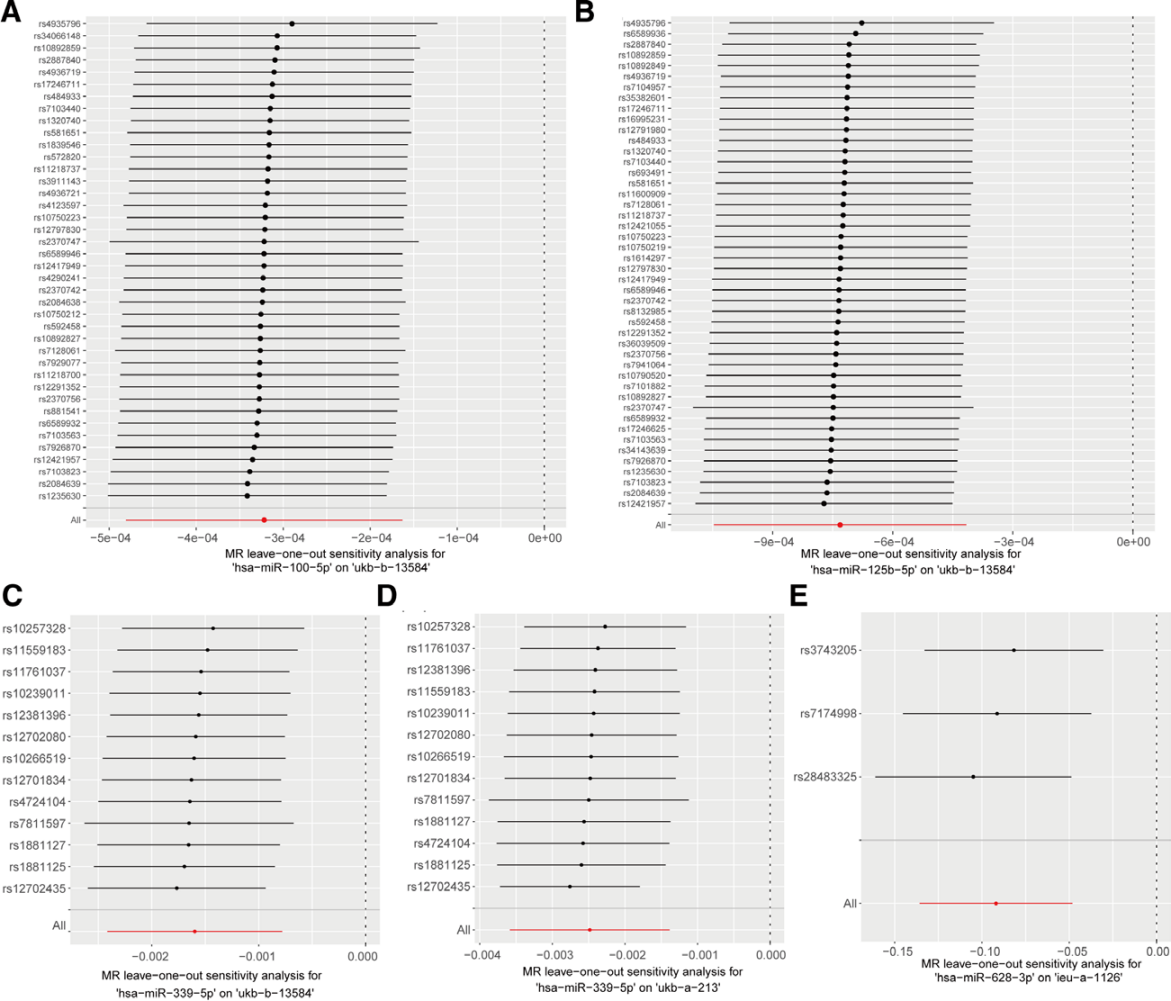
Results from leave-one-out sensitivity analysis. Results of (A) hsa-miR-100-5p, (B) hsa-miR-125b-5p, and (C) hsa-miR-339-5p on ukb-b-13584 (MRC-IEU consortium, 35,102 cases/388,356 controls). (D) Results of hsa-miR-339-5p on ukb-a-213 (Neale Lab consortium, 25,865 cases/283,784 controls). (E) Results of hsa-miR-628-3p on ieu-a-1126 (BCAC consortium, 122,977 cases/controls). Sensitivity assessed by sequentially excluding each SNP using IVW method; no single SNP significantly altered the overall causal estimates. BCAC = Breast Cancer Association Consortium, IVW = inverse-variance weighted, SNP = single nucleotide polymorphism.

In the MRC-IEU consortium, IVW analysis suggested that 3 miRNAs, hsa-miR-100-5p, hsa-miR-125b-5p, and hsa-miR-339-5p, were significantly associated with reduced breast cancer risk (Fig. 2A). In the Neale Lab consortium, hsa-miR-339-5p showed a significant association with decreased breast cancer risk (Fig. 2B). In the Breast Cancer Association Consortium, hsa-miR-628-3p was found to be significantly protective against breast cancer (Fig. 2C). To assess the robustness of these results, heterogeneity was evaluated using Cochran *Q* statistic from the IVW method, with all *Q P*-values > .05 (*Q* ranging from 0.7434 to 40.1540 across miRNAs), indicating no significant heterogeneity. Additionally, the MR-Egger intercept test was conducted to detect directional horizontal pleiotropy, revealing intercept values close to zero (ranging from −0.0005 to 0.0006) and all *P*-values > .05 (Table 1), suggesting no evidence of directional pleiotropy. Leave-one-out sensitivity analysis results are shown in Figure 3.

**Table 1:** Pleiotropy and heterogeneity tests in the discovery cohort.

Consortium	Exposure	Pleiotropy			Heterogeneity		
		Egger intercept	SE	*P*-value	*Q*	*Q*_df	*Q*_*P*val
MRC-IEU	hsa-miR-100-5p	0.0002	0.0002	.3076	33.8242	39	.7046
MRC-IEU	hsa-miR-125b-5p	0.0001	0.0002	.6204	40.1540	45	.6770
MRC-IEU	hsa-miR-339-5p	0.0005	0.0006	.4140	10.0453	12	.6120
Neale Lab	hsa-miR-339-5p	0.0006	0.0008	.4340	16.2192	12	.1814
BCAC	hsa-miR-628-3p	−0.0005	0.0404	.9928	0.7434	2	.6896

BCAC = Breast Cancer Association Consortium, SE = standard error.

### 3.3. Identification of candidate causal miRNA targets for breast cancer

**Figure 4.**
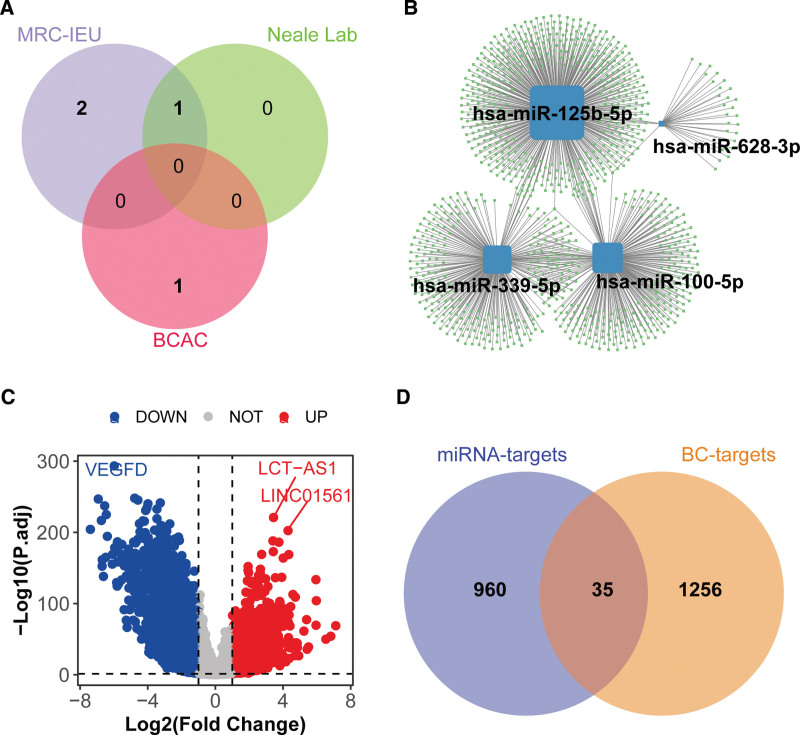
Identification of serum-circulating miRNA targets associated with breast cancer. (A) Causal miRNAs identified by integrating 3 breast cancer GWAS datasets (MRC-IEU: 35,102 cases/388,356 controls; Neale Lab: 25,865 cases/283,784 controls; BCAC: 122,977 cases/controls). (B) Regulatory network of causal miRNAs and their experimentally validated targets (from miRTarBase v9.0^[19]^; 995 unique targets across 4 miRNAs). (C) Volcano plot depicting differentially expressed genes in the TCGA-BRCA cohort (1113 breast cancer cases/113 healthy controls; analyzed using limma package, adjusted *P* < .05, log2 (fold change) > 1; 3347 upregulated and 4562 downregulated genes). (D) Venn diagram illustrating the overlap between miRNA targets and breast cancer-associated survival genes (1291 DEGs identified via univariate Cox regression, *P* < .05). BCAC = Breast Cancer Association Consortium.

Through the intersection of 3 independent consortia, we identified 4 causal miRNAs (Fig. 4A). Experimentally validated targets of these miRNAs were screened using miRTarBase v9.0, yielding 995 unique targets: hsa-miR-125b-5p (494 targets), hsa-miR-100-5p (264 targets), hsa-miR-339-5p (244 targets), and hsa-miR-628-3p (31 targets) (Fig. 4B). Differential expression analysis in the TCGA-BRCA cohort revealed 3347 significantly upregulated genes and 4562 downregulated genes (adjusted *P* < .05, log2(fold change) > 1; Fig. 4C). Univariate Cox regression analysis identified 1291 DEGs significantly associated with overall survival in breast cancer (*P* < .05; Table S2, Supplemental digital content, https://links.lww.com/MD/R299). Finally, overlapping miRNA targets with survival-associated DEGs identified 36 candidate causal miRNA targets potentially mediating breast cancer progression (Fig. 4D).

### 3.4. Pathways and biological processes mediated by causal miRNAs in breast cancer

**Figure 5.**
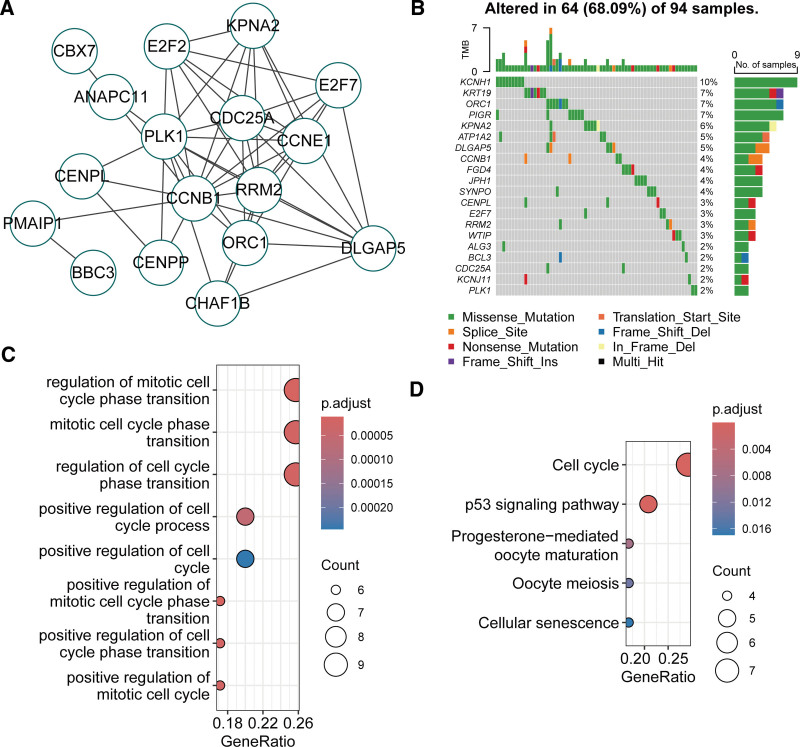
Somatic mutation and functional enrichment analyses of causal miRNA targets in breast cancer. (A) Protein–protein interaction network of miRNA targets (17 nodes, 52 edges; constructed using Cytoscape v3.10.2). (B) Oncoplot displaying somatic mutation profiles of miRNA targets in the TCGA-BRCA cohort (1113 breast cancer cases; analyzed using maftools package; mutation frequencies shown, with KCNJ1 exhibiting the highest frequency). (C) Gene Ontology terms and (D) KEGG pathways were significantly enriched among candidate targets (36 overlapping genes; analyzed using ClusterProfiler v4.12.6^[20]^; FDR < 0.05 for enrichment significance). FDR = false discovery rate, KEGG = Kyoto Encyclopedia of Genes and Genomes.

The protein–protein interaction network of causal miRNA targets comprised 17 nodes and 52 edges, highlighting robust functional interactions among these genes (Fig. 5A). Somatic mutation analysis revealed aberrant mutation patterns in these targets within the TCGA-BRCA cohort, with KCNJ1 being the most frequently mutated gene (Fig. 5B). Functional enrichment analysis demonstrated that these targets were strongly associated with cell cycle regulation (Fig. 5C). Kyoto Encyclopedia of Genes and Genomes pathway analysis further implicated their roles in cell cycle, p53 signaling pathway, progesterone-mediated oocyte maturation, oocyte meiosis, and cellular senescence (Fig. 5D). These findings suggest that causal miRNAs may attenuate breast cancer risk by modulating p53 signaling and cell cycle progression.

### 3.5. Independent cohort validation of hsa-miR-339-5p

Subsequently, we used the Nikpay, M et al cohort to validate the 4 candidate causal miRNAs of breast cancer. However, hsa-miR-628-3p was not covered by the validation set. IVW analysis showed (Table 2) that hsa-miR-339-5p has a protective effect against breast cancer in ukb-b-13584 (odds ratio [OR]: 0.994, 95% confidence interval: 0.99–0.999). Additionally, hsa-miR-339-5p was also observed to have a protective effect against breast cancer in ukb-a-213 (OR: 0.99, 95% confidence interval: 0.985–0.995). In contrast, hsa-miR-100-5p and hsa-miR-125b-5p did not show significant protective associations in the validation cohort (*P* > .05 for both), limiting their confirmation to the discovery phase.

**Table 2:** Validation of miRNA biomarkers for breast cancer using independent miRNA eQTL data.

Outcome	Exposure	Method	nSNPs	Beta	SE	*P*-value	OR (95% CI)
ukb-b-13584	hsa-miR-100-5p	MR-Egger	10	−0.00357	0.00930	.71159	0.996 (0.978–1.015)
ukb-b-13584	hsa-miR-100-5p	IVW	10	−0.00039	0.00335	.90696	1 (0.993–1.006)
ukb-b-13584	hsa-miR-125b-5p	MR-Egger	12	−0.00340	0.00856	.69944	0.997 (0.98–1.013)
ukb-b-13584	hsa-miR-125b-5p	IVW	12	−0.00496	0.00325	.12677	0.995 (0.989–1.001)
ukb-b-13584	hsa-miR-339-5p	MR-Egger	23	−0.00631	0.00693	.37314	0.994 (0.98–1.007)
ukb-b-13584	hsa-miR-339-5p	IVW	23	−0.00572	0.00227	.01170	0.994 (0.99–0.999)
ukb-a-213	hsa-miR-339-5p	MR-Egger	23	−0.01252	0.00817	.14041	0.988 (0.972–1.004)
ukb-a-213	hsa-miR-339-5p	IVW	23	−0.01029	0.00263	.00009	0.99 (0.985–0.995)

IVW = inverse-variance weighted.

### 3.6. Pan-cancer analysis of causal miRNAs and their targets

We further investigated the pan-cancer relevance of causal miRNAs and their targets. Expression profiling revealed aberrant levels of causal miRNAs across multiple cancer types. Notably, hsa-miR-125b-5p and hsa-miR-100-5p were significantly downregulated in most cancers (Table S3, Supplemental digital content, https://links.lww.com/MD/R299), though no significant dysregulation was observed in breast cancer tissues (Table 3). Prognostic analysis demonstrated that all 4 causal miRNAs were associated with survival outcomes in pan-cancer cohorts, with hsa-miR-125b-5p exhibiting a protective effect on overall survival in multiple malignancies (Table S4, Supplemental Digital Content, https://links.lww.com/MD/R299). However, no significant association between causal miRNAs and breast cancer prognosis was detected (Table 4). Strikingly, significant negative correlations were observed between causal miRNAs and their target genes in breast cancer tissues (Table 5), further supporting their functional relevance in breast carcinogenesis.

**Table 3:** Expression analysis of causal miRNA in breast invasive carcinoma.

miRNA	CancerNum	NormalNum	CancerExp	NormalExp	FoldChange	*P*-value	FDR
hsa-miR-125b-5p	1085	104	723.36	2660.52	0.27	1.50E−70	2.10E−68
hsa-miR-628-3p	1085	104	0.76	0.62	1.23	.21	0.39
hsa-miR-339-5p	1085	104	47.51	50.39	0.94	.14	0.3
hsa-miR-100-5p	1085	104	3036.72	10772.41	0.28	3.10E−68	4.00E−66

FDR = false discovery rate.

**Table 4:** Univariate Cox regression analysis of causal miRNA in breast invasive carcinoma.

miRNA	CancerNum	Median	Coef	HR	*P*-value
hsa-miR-125b-5p	1082	572.88	−0.13	0.88	.44
hsa-miR-628-3p	1082	0.47	0.01	1.01	.95
hsa-miR-339-5p	1082	35.4	0.03	1.03	.86
hsa-miR-100-5p	1082	2473.78	−0.2	0.82	.21

HR = hazards ratio.

**Table 5:** Co-expression analysis of causal miRNA and their targets in breast invasive carcinoma.

miRNA	Targets	SampleNum	Coefficient-*R*	*P*-value
hsa-miR-125b-5p	CCNE1	1085	−0.008	7.95E−01
hsa-miR-125b-5p	CCNB1	1085	−0.197	5.93E−11
hsa-miR-125b-5p	SAMD10	1085	−0.134	9.38E−06
hsa-miR-125b-5p	CDC25A	1085	−0.155	2.74E−07
hsa-miR-125b-5p	BBC3	1085	−0.126	3.12E−05
hsa-miR-125b-5p	CENPP	1085	−0.039	1.96E−01
hsa-miR-125b-5p	E2F7	1085	−0.127	2.64E−05
hsa-miR-125b-5p	PMAIP1	1085	−0.052	8.76E−02
hsa-miR-125b-5p	PTH1R	1085	0.439	2.43E−52
hsa-miR-125b-5p	ACADS	1085	−0.047	1.23E−01
hsa-miR-125b-5p	KRT19	1085	−0.16	1.15E−07
hsa-miR-125b-5p	PSAT1	1085	0.191	2.09E−10
hsa-miR-125b-5p	BCL3	1085	−0.085	5.11E−03
hsa-miR-125b-5p	PIGR	1085	0.263	1.30E−18
hsa-miR-125b-5p	GJB7	1085	0.065	3.17E−02
hsa-miR-125b-5p	CBX7	1085	0.051	9.46E−02
hsa-miR-125b-5p	E2F2	1085	−0.149	8.03E−07
hsa-miR-339-5p	KCNJ11	1085	−0.127	2.87E−05
hsa-miR-339-5p	SYNPO	1085	−0.234	6.09E−15
hsa-miR-339-5p	ZNF703	1085	−0.135	7.75E−06
hsa-miR-339-5p	FGD4	1085	−0.02	5.08E−01
hsa-miR-339-5p	KCNH1	1085	−0.018	5.52E−01
hsa-miR-339-5p	ORC1	1085	0.165	4.53E−08
hsa-miR-339-5p	CHAF1B	1085	0.181	1.90E−09
hsa-miR-339-5p	DLGAP5	1085	0.207	5.78E−12
hsa-miR-339-5p	CENPL	1085	0.154	3.25E−07
hsa-miR-100-5p	WTIP	1085	0.364	2.99E−35
hsa-miR-100-5p	KPNA2	1085	−0.221	2.03E−13
hsa-miR-100-5p	ATP1A2	1085	0.272	7.75E−20
hsa-miR-100-5p	ALG3	1085	−0.22	2.40E−13
hsa-miR-100-5p	RRM2	1085	−0.219	2.74E−13
hsa-miR-100-5p	JPH1	1085	−0.088	3.83E−03
hsa-miR-100-5p	MMP1	1085	−0.072	1.77E−02
hsa-miR-100-5p	ANAPC11	1085	−0.167	3.37E−08
hsa-miR-100-5p	PLK1	1085	−0.185	8.20E−10

## 4. Discussion

This study utilized MR analysis to investigate the potential causal relationship between miRNAs and breast cancer, revealing that several miRNAs may exert a protective effect in the development of breast cancer. These findings not only provide novel biomarkers for early prevention and intervention strategies against breast cancer but also offer significant insights into the molecular mechanisms underlying its pathogenesis.

Our study has identified significant associations between several miRNAs – namely hsa-miR-100-5p, hsa-miR-125b-5p, hsa-miR-339-5p, and hsa-miR-628-3p – and a reduced risk of breast cancer. Among these, hsa-miR-339-5p emerged as the primary candidate, as it was the only miRNA validated in an independent cohort, consistently showing a protective effect against breast cancer (OR: 0.994 in ukb-b-13584 and 0.99 in ukb-a-213). Exosome-derived hsa-miR-339-5p has been identified as a potential biomarker for bone metastasis in prostate cancer.^[21]^ In breast cancer, hsa-miR-339-5p is downregulated; however, its expression does not correlate with TNM staging.^[22]^ Interestingly, in glioblastoma multiforme tumor tissues, hsa-miR-339-5p is significantly upregulated.^[23]^ Additionally, studies have demonstrated that hsa-miR-339-5p plays a crucial role in the regulation of lung cancer and may serve as a biomarker to predict lung cancer progression.^[24]^ These findings reinforce the promise of hsa-miR-339-5p as both a biomarker and therapeutic target, particularly given its validation across cohorts in our study.

As secondary or exploratory findings, we also observed protective associations for hsa-miR-100-5p and hsa-miR-125b-5p in the initial MR analyses. Specifically, the high expression of hsa-miR-100-5p has been shown to improve the prognosis in hepatocellular carcinoma patients within Asian populations by targeting PLK1.^[25]^ Additionally, plasma levels of hsa-miR-100-5p are significantly elevated in breast cancer patients and correlate with disease staging, demonstrating its potential utility in diagnosing breast cancer and early-stage breast cancer.^[26]^ However, the role of hsa-miR-100-5p appears to be context-dependent. For instance, in pancreatic ductal adenocarcinoma, hsa-miR-100-5p has been reported to promote cancer cell growth,^[27]^ whereas it is notably downregulated in gastric cancer compared with normal tissue, yet within tumor samples, higher expression levels of hsa-miR-100-5p are associated with poorer overall survival and disease-free survival.^[28]^ In oral cancer, elevated levels of hsa-miR-100-5p have been linked to extracapsular extension and worse survival rates.^[29]^ Furthermore, in esophageal cancer, hsa-miR-100-5p exhibits low expression, but its high expression correlates with better patient outcomes.^[30]^ Interestingly, in aggressive forms of bladder urothelial carcinoma, hsa-miR-100-5p expression is lower compared with nonaggressive cases.^[31]^ These findings collectively suggest that the differential expression of hsa-miR-100-5p across various types of cancer may confer diverse functional roles, highlighting the complexity of its involvement in different malignancies.

Hsa-miR-125b-5p, a member of the miR-125 family, has already emerged as a significant biomarker for cancer diagnosis, treatment, and prognosis.^[32]^ Recent studies have demonstrated that the overexpression of miR-125b can promote colorectal cancer development in patients with primary sclerosing cholangitis, primary sclerosing cholangitis-ulcerative colitis, and ulcerative colitis alone by influencing the sphingosine-1-phosphate (S1P)/ceramide axis and interacting with genes related to SPHK2 and glycolytic pathways. This effect is particularly pronounced in cancers characterized by high microsatellite instability.^[33]^

In breast cancer, specifically in Luminal A subtype, hsa-miR-125b-5p serves as a potential diagnostic biomarker and regulator.^[34]^ However, its expression levels vary significantly across different types of cancer. For instance, hsa-miR-125b-5p is downregulated in hepatocellular carcinoma^[35]^ and cervical cancer, where it exhibits markedly lower expression in malignant samples compared with normal controls.^[36]^ Additionally, its expression is reduced in prostate cancer^[37]^ and colorectal cancer, where decreased levels are associated with favorable prognosis and could be utilized for early diagnosis and prognostic assessment.^[38,39]^ The mechanism underlying its role in colorectal cancer involves regulation by MTUS1.^[40]^ Furthermore, research by Chang et al indicated that hsa-miR-125b-5p is associated with macrophages M0 and T follicular helper cells, playing an active role in the metastasis of colorectal adenocarcinoma.^[41]^ In non-small cell lung cancer, hsa-miR-125b-5p is downregulated during immunotherapy, which may lead to enhanced T-cell function and improved responses to treatment.^[42]^ While these secondary miRNAs show promising associations in our MR analyses, their lack of independent validation limits their emphasis compared with hsa-miR-339-5p.

A potential disconnect exists between the MR findings, which suggest a protective effect of these miRNAs (particularly hsa-miR-339-5p) based on genetic predisposition, and the observed lack of significant dysregulation or prognostic associations in breast cancer tissues from pan-cancer analyses. It is important to note that MR-detected effects primarily reflect genetically determined lifelong exposure to altered miRNA levels, which may influence cancer risk at a population level but not necessarily manifest as altered expression in established tumors due to tumor heterogeneity, epigenetic modifications, or compensatory mechanisms. Despite the absence of significant clinical associations in breast cancer-specific prognosis or tissue expression, the functional relevance is supported by significant negative correlations between these miRNAs and their target genes in breast tumors (Table 5), indicating potential regulatory roles in carcinogenesis.

To elucidate the potential mechanisms by which these causal miRNAs influence breast cancer, we investigated their target genes and associated pathways. The candidate causal miRNA targets were enriched in cell cycle regulation and the p53 signaling pathway (Fig. 5C and D), which are biologically plausible in the context of miRNA-mediated regulation. The p53 pathway is a central tumor suppressor mechanism that responds to cellular stress by inducing cell cycle arrest, apoptosis, or senescence, thereby preventing malignant transformation.^[43]^ MiRNAs such as hsa-miR-339-5p, hsa-miR-100-5p, and hsa-miR-125b-5p can modulate this pathway by targeting key regulators like PLK1 (for hsa-miR-100-5p) or other downstream effectors, potentially enhancing p53 activity and inhibiting breast cancer progression. Similarly, cell cycle regulation is critical for controlling proliferation, and dysregulation leads to unchecked growth in cancers such as breast cancer.^[44]^ Our identified miRNAs likely attenuate risk by repressing genes involved in G1/S transition or mitotic progression, as evidenced by the enrichment in progesterone-mediated oocyte maturation and cellular senescence pathways, which share molecular overlaps with cancer cell cycle control. Synthesizing these findings, we propose a mechanistic hypothesis: genetically elevated levels of these protective miRNAs (led by hsa-miR-339-5p) converge on the p53-cell cycle axis to suppress oncogenic signaling, reduce proliferation, and promote apoptosis in breast epithelial cells, thereby lowering overall cancer risk. This model integrates the MR causal inferences with target gene correlations and pathway enrichments, suggesting that miRNA-based interventions could target this axis for prevention or therapy.

While the nonsignificant MR-Egger intercepts and lack of heterogeneity support the robustness of our causal estimates, these do not completely rule out pleiotropy or heterogeneity, especially considering the limited number of SNPs per miRNA (at least 3, but potentially reducing statistical power for detection). However, this study has several limitations. First, despite validation across multiple independent cohorts, the predominantly European ancestry of the study population may limit the generalizability of our findings. Future studies should incorporate more diverse ethnic and geographic populations to enhance external validity. Second, while rigorous statistical adjustments for multiple testing and potential confounders were applied, the causal roles of these miRNAs require confirmation in larger-scale clinical cohorts. Finally, systematic functional studies (e.g., in vitro and in vivo experiments) are warranted to validate the biological impact and molecular mechanisms of serum-circulating miRNAs on breast cancer pathogenesis.

## 5. Conclusion

In summary, this study identified multiple serum-circulating miRNAs associated with breast cancer risk and elucidated their candidate targets and regulatory pathways, providing a theoretical foundation for early prevention and targeted intervention in breast cancer. These findings highlight their translational potential for developing personalized risk prediction models and preventive strategies. Future studies should prioritize functional validation of these miRNAs and assess their clinical utility as noninvasive biomarkers or therapeutic targets in breast cancer.

## Author contributions

**Conceptualization:** Jianlin Ren, Lifeng Zhu.

**Data curation:** Jianlin Ren, Lifeng Zhu.

**Formal analysis:** Jianlin Ren, Lifeng Zhu.

**Funding acquisition:** Jianlin Ren, Lifeng Zhu.

**Investigation:** Jianlin Ren, Lifeng Zhu.

**Methodology:** Jianlin Ren, Lifeng Zhu.

**Project administration:** Jianlin Ren, Lifeng Zhu.

**Resources:** Jianlin Ren, Lifeng Zhu.

**Software:** Jianlin Ren, Lifeng Zhu.

**Supervision:** Jianlin Ren, Lifeng Zhu.

**Validation:** Jianlin Ren, Lifeng Zhu.

**Visualization:** Jianlin Ren, Lifeng Zhu.

**Writing – original draft:** Jianlin Ren, Lifeng Zhu.

**Writing – review & editing:** Lifeng Zhu.

## Supplementary Material


